# Acid ceramidase promotes senescent cell survival

**DOI:** 10.18632/aging.203170

**Published:** 2021-06-08

**Authors:** Rachel Munk, Carlos Anerillas, Martina Rossi, Dimitrios Tsitsipatis, Jennifer L. Martindale, Allison B. Herman, Jen-Hao Yang, Jackson A. Roberts, Vijay R. Varma, Poonam R. Pandey, Madhav Thambisetty, Myriam Gorospe, Kotb Abdelmohsen

**Affiliations:** 1 Laboratory of Genetics and Genomics, National Institute on Aging-Intramural Research Program, National Institutes of Health, Baltimore, Maryland 21224, USA; 2 Laboratory of Behavioral Neuroscience, National Institute on Aging-Intramural Research Program, National Institutes of Health, Baltimore, Maryland 21224, USA

**Keywords:** senotherapy, post-transcriptional, SASP, senescent cell metabolism, translational control

## Abstract

Cellular senescence is linked to chronic age-related diseases including atherosclerosis, diabetes, and neurodegeneration. Compared to proliferating cells, senescent cells express distinct subsets of proteins. In this study, we used cultured human diploid fibroblasts rendered senescent through replicative exhaustion or ionizing radiation to identify proteins differentially expressed during senescence. We identified acid ceramidase (ASAH1), a lysosomal enzyme that cleaves ceramide into sphingosine and fatty acid, as being highly elevated in senescent cells. This increase in ASAH1 levels in senescent cells was associated with a rise in the levels of *ASAH1* mRNA and a robust increase in ASAH1 protein stability. Furthermore, silencing ASAH1 in pre-senescent fibroblasts decreased the levels of senescence proteins p16, p21, and p53, and reduced the activity of the senescence-associated β-galactosidase. Interestingly, depletion of ASAH1 in pre-senescent cells sensitized these cells to the senolytics Dasatinib and Quercetin (D+Q). Together, our study indicates that ASAH1 promotes senescence, protects senescent cells, and confers resistance against senolytic drugs. Given that inhibiting ASAH1 sensitizes cells towards senolysis, this enzyme represents an attractive therapeutic target in interventions aimed at eliminating senescent cells.

## INTRODUCTION

Senescence is a phenotype associated with indefinite growth arrest of the cell, first observed by Hayflick [1]. Senescence can be induced by telomere shortening, genomic injury, epigenomic damage, signaling from oncoproteins, and other sublethal stresses. Senescent cells can also be identified by morphological changes including an enlarged and flattened shape, the presence of vacuoles, and irregular-shaped nuclei [2]. Biochemical changes include DNA damage, increased levels of TP53 (p53), CDKN1A (p21), and CDKN2A (p16), and increased function of a senescence-associated β-galactosidase (SA-βGal) active at pH 6 [3, 4]. Although senescent cells do not divide, they remain metabolically active [5, 6]; this metabolic profile includes enhanced lysosomal activity associated with both growth arrest and a senescence-associated secretory phenotype (SASP), whereby many proteins are secreted, including interleukin (IL) 6 and IL8 [7, 8]. Lysosomal dysfunction is also linked to mitochondrial turnover and autophagy, leading to an increase in reactive oxygen species (ROS) and altered metabolic activities [9].

Senescence can be beneficial in young organisms by enabling tissue remodeling, embryonic development, wound healing, and suppression of tumorigenesis [10–14]. However, it can be detrimental in old organisms as senescent cells accumulate over time and promote premature aging and aging-associated conditions such as atherosclerosis, liver fibrosis, insulin resistance, neurodegenerative disorders, chronic inflammation, and cancer [10, 15–17]. Accordingly, some age-associated diseases were found to improve through the clearance of senescent cells using genetic approaches or senolytic drugs [18, 19]. In mouse models, first-generation senolytics were used successfully to clear senescent cells in atherosclerosis and neurodegeneration. Ongoing studies in humans employ targeted senolytics for the treatment of eye diseases and osteoarthritis [20]. The transgene INK-ATTAC was used in mouse models for the inducible clearance of p16-positive senescent cells leading to an improvement in age-associated pathologies such as sarcopenia, loss of adiposity, cataracts, cardiac hypertrophy, kidney disease, cancer, atherosclerosis, osteoarthritis, and neurodegeneration [21]. The clearance of senescent cells using senolytic drugs was similarly beneficial towards cardiovascular disease, neurodegeneration, obesity, cancer, type 2 diabetes, sarcopenia, and osteoarthritis [22–24].

Given that senescent cells accumulate with age and promote age-related diseases, there is growing interest in identifying factors that can eliminate senescent cells more efficiently. Here, we used transcriptomic and proteomic analyses to identify such factors. Our studies revealed robustly increased expression levels of the lysosomal enzyme ASAH1 (N-acylsphingosine amidohydrolase 1; acid ceramidase) in WI-38 human diploid fibroblasts rendered senescent by either replicative exhaustion or exposure to ionizing radiation (IR). ASAH1 catalyzes the conversion of ceramide into sphingosine and fatty acid and has been associated with pathologies such as Farber’s disease, Alzheimer’s disease, cancer, diabetes, and spinal muscular atrophy [25–27]. We found a striking increase in ASAH1 protein levels in senescent cells that was independent of mRNA localization, stability, or translation; instead, ASAH1 protein was more stable in senescent cells than in proliferating cells, at least in part due to a decrease in proteasome-mediated ASAH1 degradation. Furthermore, silencing ASAH1 in pre-senescent fibroblasts decreased the levels of the senescence markers p16, p21 and p53, and also reduced the activity of the SA-βGal. Interestingly, selective inhibition of ASAH1 using Acid Ceramidase Inhibitor IV (ACi) enhanced death of senescent cells but not proliferating cells and was further exacerbated by addition of the senolytics Dasatinib and Quercetin (D+Q). This decrease in cell viability was linked to an increase in caspase activity. Similarly, silencing ASAH1 sensitized pre-senescent cells to D+Q, likely by reprogramming them to death through apoptosis. Together, our findings suggest that ASAH1 promotes senescence and senescent-cell resistance to senolytics, underscoring its possible use in senotherapy.

## MATERIALS AND METHODS

### Cell culture, replicative senescence, and SA-βGal assays

WI-38 human diploid fibroblasts (HDFs) were cultured in Dulbecco's modified Eagle's medium (DMEM, Gibco) supplemented with 20% heat-inactivated fetal bovine serum (FBS, Gibco), antibiotics penicillin and streptomycin (Gibco), and non-essential amino acids (Gibco) in a 5% CO_2_ incubator. Primary human coronary artery vascular smooth muscle cells (VSMCs) were obtained as cryopreserved secondary cultures from LifeLine Cell Technology and maintained in VascuLife® SMC Medium Complete Kit from LifeLine Cell Technology following the manufacturer’s protocol.

Proliferating WI-38 cells were used at low population doubling levels (PDL) ranging between PDL20 and PDL24. Replicative senescence was achieved by passaging WI-38 cells in culture until their growth rate decreased indicating that a senescent state had been attained (PDL48 to PDL52). Senescence-associated β-galactosidase (SA-βGal) activity in WI-38 cells was assessed following the manufacturer’s protocol (Cell Signaling Technology). Cellular senescence was quantified using SPiDER-βGal analysis following the manufacturer’s protocol (Dojindo Molecular Technologies). Briefly, cells were seeded overnight at a density of 2.5 × 10^4^ in triplicate in a 96-well plate. The following day, cells were counted using the Cell Count normalization assay following the manufacturer’s protocol (Dojindo Molecular Technologies). Fluorescence was measured using a Perkin Elmer Victor plate reader at excitation 350 nm, emission 461 nm. The same plate was then used for the SPiDER-βGal assay. Cells were lysed at 25° C for 10 min; 50 μl of SPiDER-βGal working solution was added to each well and incubated at 37° C, 5% CO_2_ for 60 min; 100 μl of Stop Solution was then added to each well. Fluorescence was measured using a Promega Glomax Explorer at 520 nm excitation, 580-620 nm emission. Normalized SA-βGal activity was calculated by this formula: (Fluorescent intensity of SA-βGal activity) / (Fluorescent intensity of cell number – Fluorescent intensity of blank).

### Transfection and treatments

Pre-senescent (PDL38 to PDL45) WI-38 fibroblasts were seeded in triplicate overnight at 2.5×10^5^ cells/well in a 6-well tissue culture plate for ~50% confluence by the next day. Cells were transfected using Lipofectamine 2000 (Thermo Fisher) in 1 ml Optimem (Gibco) following the manufacturer’s protocol. Control siRNA (sc-37007) or ASAH1 siRNA (sc-105032) were used at 35 nM for 48 h. Proliferating (P, PDL20-24) and replicatively senescent (S, PDL48-52) cells were treated with either DMSO (control vehicle) or the inhibitor of RNA polymerase II Actinomycin D (Sigma) (2.5 μg/ml) followed by RNA isolation to test mRNA stability. For ionizing radiation (IR)-induced senescence, proliferating (PDL20-24) WI-38 cells were exposed to 10 Gy and harvested 10 days later. For cycloheximide (CHX) treatments, proliferating (PDL20-24) and replicative senescent (PDL48-52) cells were treated with either DMSO (control vehicle) or 50 μg/ml CHX for 0, 1, 2 or 4 h. For MG132 treatments, proliferating (PDL20-24) and replicative senescent (PDL48-52) cells were treated with either ethanol (control vehicle), or 10 μM MG132 for 0, 2, 4 or 6 h. Cells were treated with (ACi; Calbiochem, ACI IV, BOC, Cat# 533371) at 50 μM for 48 h.

### Protein analysis

Cell pellets were washed twice in cold PBS, the supernatant was discarded, and cells were lysed in RIPA lysis buffer (Santa Cruz Biotechnology) containing protease inhibitors (Roche) and incubated on ice for 10 min, followed by sonication for 5 min. The sonicated samples were centrifuged for 10 min at 25° C to remove the insoluble fraction. The remaining supernatant was the whole-cell lysate. Lysates were separated by SDS-polyacrylamide gel electrophoresis (SDS-PAGE) and transferred onto nitrocellulose membranes (Biorad). Incubations with primary antibodies recognizing ASAH1 (sc-136275), p21 (CDKN1A), p53 (TP53) (from Santa Cruz Biotechnology), as well as an antibody recognizing p16 (CDKN2A, from BD Biosciences), were followed by incubations with the appropriate secondary antibodies conjugated with horseradish peroxidase (GE Healthcare). The levels of proteins GAPDH, β-actin (ACTB), and HSP90 (from Santa Cruz Biotechnology), which displayed unchanged levels, were monitored to control for loading. Signals were developed using Enhanced Chemiluminescence (ECL) and acquired by KwikQuant Imaging System (Kindle Biosciences). Proteomic data are displayed in Supplementary Table 1.

### RNA isolation, sequencing, and RT-qPCR analysis

RNA was isolated from WI-38 fibroblasts using Direct-zol RNA kit following the manufacturer’s instructions (Zymo Research). RNA-seq analyses of proliferating and senescent cells (replicative or IR-induced senescence) were performed as described in [28], and the data are available at GEO accession number GSE85771 [28]. Total RNA was used for gene expression analysis by reverse transcription (RT) followed by quantitative (q)PCR analysis. RT was performed by using random hexamers and reverse transcriptase (Maxima Reverse Transcriptase, Thermo Fisher) and qPCR analysis was carried out using gene-specific primers and SYBR green master mix (Kapa Biosystems) using QuantStudio5 (Thermo Fisher). PCR primer pairs (each forward and reverse) were as follows: GGAGTTGCGTCGCCTTAGT and TGGTCCTGAAGGAGGATAGG for *ASAH1* mRNA, GTAAAGAAGCTGCCCAGCAC and GCCGGTGAGTGAGCTTGAG for *ASAH1* pre-mRNA, CATGTACGTTGCTATCCAGGC and CTCCTTAATGTCACGCACGAT for *ACTB* mRNA, CTCTGCTCCTCCTGTTCGAC and ACGACCAAATCCGTTGACTC for *GAPDH* mRNA, CGAACGTCTGCCCTATCAACTT and ACCCGTGGTCACCATGGTA for *18S* rRNA, and CCTGCCCAAGCTCTACCTT and AAGGCAGAAGATGTAGAGC for *p21* mRNA, GAGCTGGGGAATGGGACT and TGATGGCATGGACTGTGG for *GAPDH* pre-mRNA, CAAAACTCCCGTGCTGATCA and GGCTGGAGTGCAGTGGCTAT for *7SL* RNA, GCTGTGGAGTTCTTAAATATCAACC and TTCTCAATCCTGAAATCCCCTA for *MALAT1* RNA, and TTCTCTCCGTCCTCGGATTCTCTG and TCTTCTTGTTCCTCCTCAGAGTCG for *MYC* mRNA.

### Polyribosome fractionation

Cells were incubated with cycloheximide (Calbiochem; 100 μg/ml, 15 min) and the cytoplasmic lysates were prepared in polysome extraction buffer (PEB), fractionated by centrifugation through 10–50% linear sucrose gradients, and divided into 12 fractions for RT-qPCR analysis, as described [29].

### Cell cycle analysis

Pre-senescent (PDL41) WI-38 cells were transfected with Control siRNA or ASAH1 siRNA as described previously. After 48 h, cells were harvested, washed with PBS and resuspended in 0.1% Triton-X in PBS for 5 min at 25° C; after brief centrifugation, the supernatant was aspirated. The pellet was incubated with RNase A (10 μg/ml in 20 mM Tris-HCl [pH 7.5]) at 37° C for 15 min; following a brief centrifugation, the supernatant was aspirated and 200 μl of 2 μg/ml propidium iodide was added. After incubation in the dark for 10 min, flow cytometry was performed on a BD FACS Canto for cell cycle analysis.

### Caspase 3/7 activity

Apoptosis was monitored by measuring Caspase 3/7 activity using the Caspase-Glo 3/7 Assay System (Promega). Briefly, Caspase-Glo® 3/7 solution at 25° C was added directly to the cells, mixed vigorously for 30 sec, and incubated at 25° C in the dark for 30 to 180 min. Luminescence was then measured using a GloMax plate reader (Promega).

### Mass spec and targeted mass spec (metabolomics)

Cells were harvested from 10-cm dishes individually from each of these groups: proliferating, replicative senescent, senescent after IR, Control siRNA, and ASAH1 siRNA. A minimum of 7 replicates were collected for each group with a minimum of 3×10^6^ cells collected per replicate. The cells were centrifuged and washed twice in cold PBS. The supernatant was discarded, and the cell pellet was frozen at -80° C. These frozen pellets were then used for targeted metabolomics using the BIOCRATES MxP Quant 500 Kit to assay approximately 40 metabolites within the following classes: (1) Ceramides, (2) Fatty acids, and (3) Sphingomyelins. Metabolites were extracted and concentrations were measured at Biocrates Life Sciences AG (Innsbruck, Austria) in pmol/106 cells. Targeted metabolomics was performed using tandem mass spectrometry with the Biocrates MxP® Quant 500 Kit (Biocrates, Innsbruck, Austria). Briefly, metabolites were measured using liquid chromatography tandem mass spectrometry (LC-MS/MS). Lipids specifically were measured by flow injection analysis-tandem mass spectrometry (FIA-MS/MS) using a 5500 QTRAP® instrument (AB Sciex, Darmstadt, Germany) with an electrospray ionization source, and small molecules were measured by LC-MS/MS using the same 5500 QTRAP® instrument. Measurement techniques are described at https://patents.google.com/patent/EP1897014B1 and https://patents.google.com/patent/EP1875401B1. A 96-well-based sample preparation device with internal standards was used to measure metabolite concentration in each sample (predefined sample amount). Next, a phenyl isothiocyanate solution was used to derivatize select classes of metabolites, and the target analytes were extracted with an organic solvent, followed by a dilution step. The extracts were then analyzed by FIA-MS/MS and LC-MS/MS methods using multiple reaction monitoring to detect the analytes. Data were quantified using mass spectrometry software (Sciex Analyst®) and imported into Biocrates MetIDQ™ software for further analysis. Metabolite values (pmol/10^6^ cells) below the limit of detection (LOD) or above the limit of quantification (ULOQ) were excluded from analyses. A proportional odds model [30], ordered logistic model and generalization of the non-parametric Wilcoxon and Kruskal-Wallis tests, was used to compare metabolite concentrations across three comparisons: Replicative senescence vs Proliferating cell control, IR-induced senescence vs Proliferating cell control, and ASAH1 siRNA vs Control siRNA. Estimates for comparisons with minimal sample size (i.e., only 1 or 2 metabolite measurements per group) could not be accurately estimated by the model and were excluded. Log odds ratios (OR; e.g., the increased or decreased odds of a higher metabolite concentration in one group compared to the other) and results significant at p < 0.05 were indicated. Complete analysis results are included in Supplementary Table 2.

## RESULTS

### ASAH1 levels increase in senescent cells

**Figure 1:**
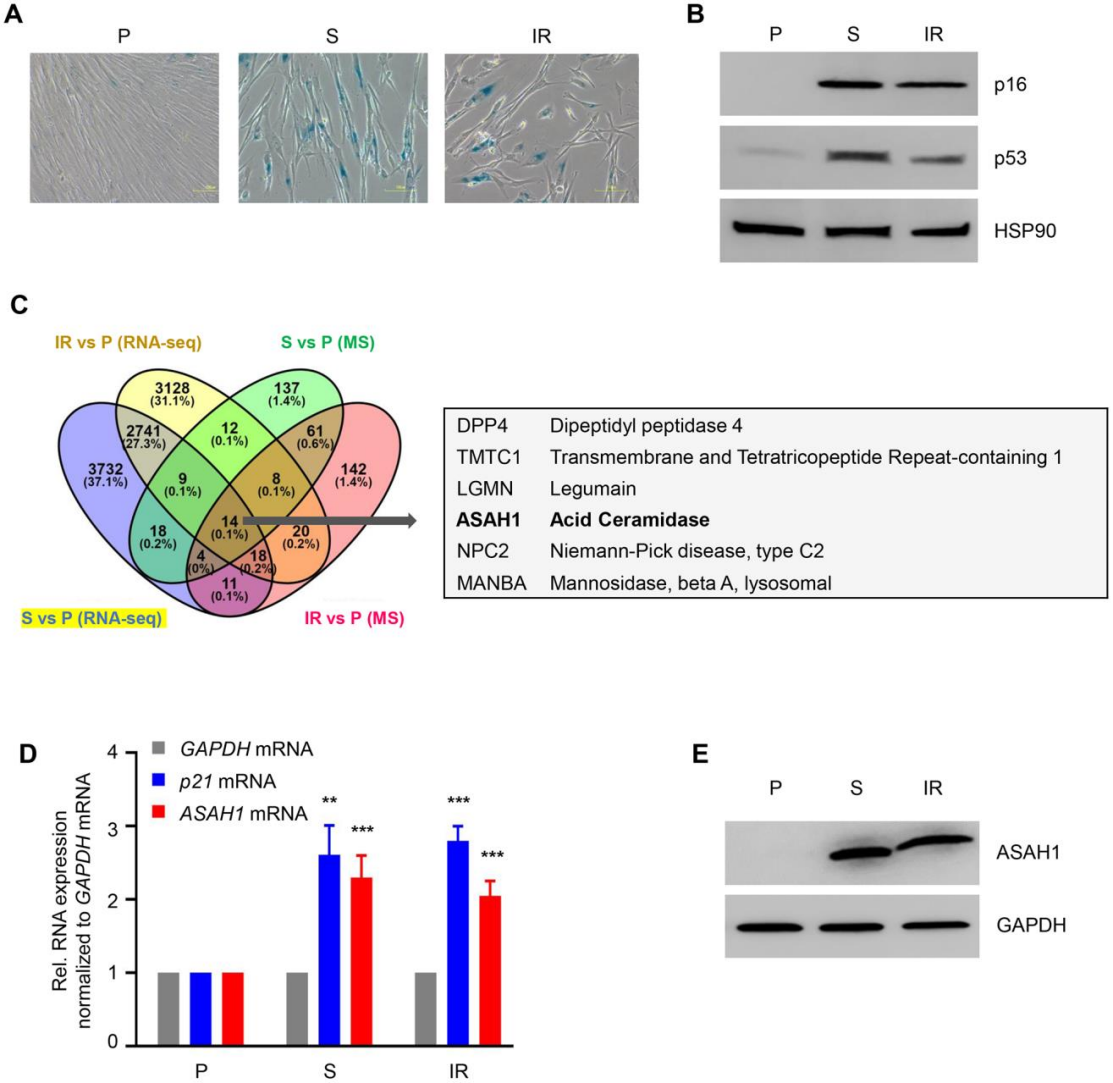
**ASAH1 is highly expressed in senescent cells.** (**A**) Senescence-associated β-galactosidase (SA-βGal) staining of WI-38 fibroblasts that were proliferating (P, PDL25) or were rendered senescent (S, PDL52) by extended culture or by exposure to ionizing radiation (IR, 10 Gy) and assayed 10 days later. (**B**) Western blot analysis of senescence marker proteins p16 and p53 in cells prepared as described in (**A**). The levels of loading control HSP90 were also assessed. (**C**) *Left*, Venn diagram showing commonly upregulated mRNAs (RNA-sequencing data) and proteins obtained by mass spec (MS) analysis (full list provided as Supplementary Table 1) in replicative senescence (S) and IR-induced senescence relative to proliferating (P) fibroblasts. *Right*, partial list of the shared mRNAs and proteins upregulated in senescent cells. (**D**, **E**) In cells that were processed as in (**A**), the steady-state levels of *p21* and *ASAH1* mRNAs were quantified by RT-qPCR analysis (mRNA levels were normalized to *GAPDH* mRNA levels), and the levels of ASAH1 were assessed by Western blot analysis (loading control protein GAPDH was included). Data in (**D**) are the means ±S.D. of three biological replicates; data in (**A**, **B**, **E**) are representative of three biological replicates. RNA-seq is provided in GSE85771.

We used proliferating (‘P’, early population doubling level, PDL22) primary human WI-38 fibroblasts to obtain senescent cells either by replicative exhaustion (‘S’, PDL52) or by treatment with ionizing radiation (‘IR’, 10 Gy followed by culture for 10 additional days). Senescence was assessed by monitoring SA-βGal activity and the levels of senescence-associated markers p16 and p53 in both models (Figure 1A, 1B; Supplementary Figure 1A). To assess the changes in protein levels, whole-cell protein lysates prepared from proliferating and senescent cells were subjected to mass spectrometry (MS) proteomic analysis (Supplementary Table 1). We also extracted whole-cell RNA from proliferating and senescent cells and performed RNA-seq analysis using an Illumina HiSeq 4000 instrument at a depth of 75 million unidirectional reads for a total of 150 million paired-end reads per sample (GEO number GSE85771) [28]. In this analysis, we compared upregulated proteins and RNAs in replicative and IR-induced senescence (Figure 1C; Supplementary Figure 1B). We observed that both *DPP4* mRNA and DPP4 protein were highly expressed, as we previously reported [31]. In addition, we observed that acid ceramidase (ASAH1) was upregulated in this comparison at both the protein and mRNA levels (Figure 1C). We also detected increased expression of ASAH1 upon doxorubicin-induced senescence in vascular smooth muscle cells (VSMCs) (Supplementary Figure 1C). The increase in *ASAH1* mRNA levels in replicative (S) and IR-induced senescence was validated by RT-qPCR analysis. These senescent cells also displayed elevated levels of *p21* mRNA (Figure 1D). Western blot analysis showed a robust increase in ASAH1 expression in both models of cell senescence (Figure 1E). In sum, ASAH1 protein levels increase strongly in senescent fibroblasts.

### Altered ceramide metabolites in senescent cells

**Figure 2:**
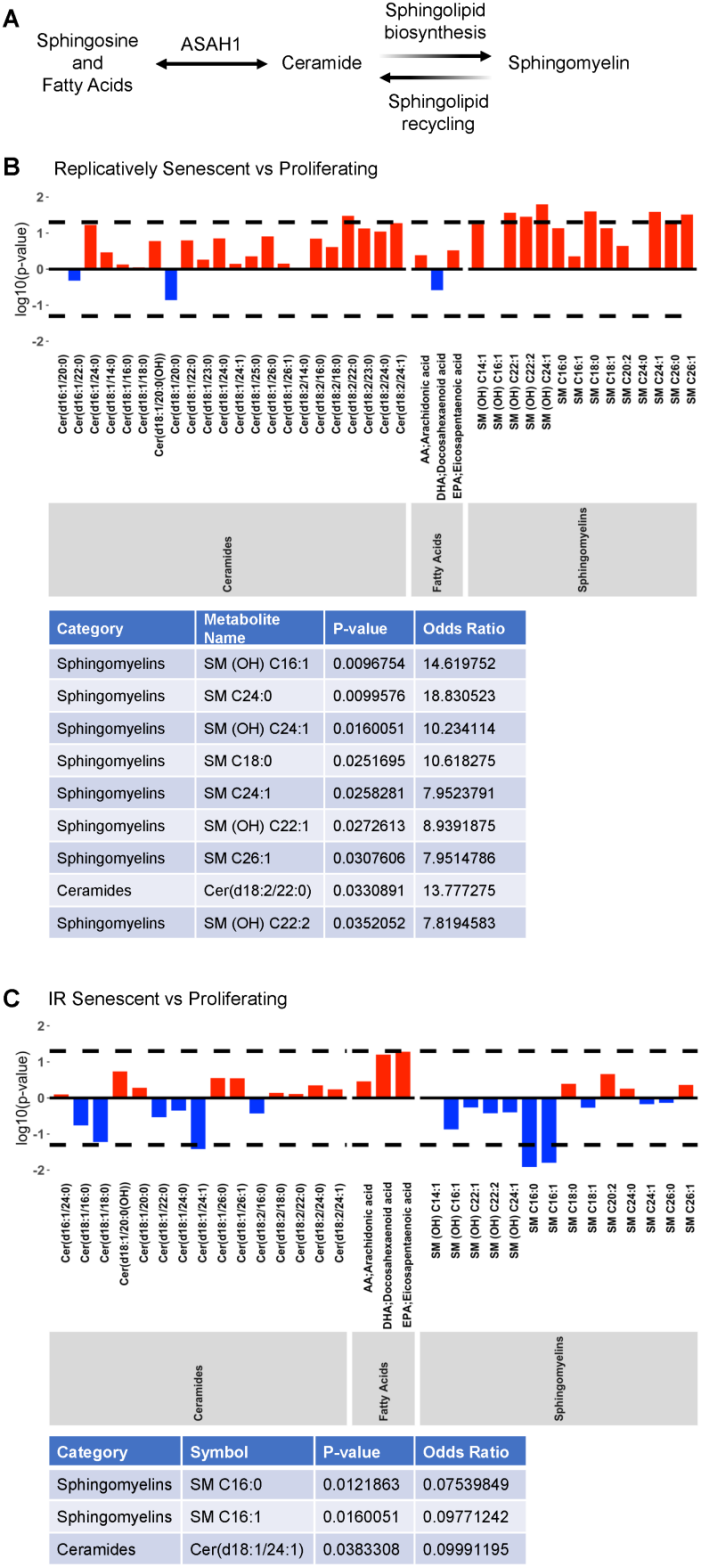
**Altered ASAH1-related metabolites in senescence.** (**A**) Schematic representation of the metabolic pathway of ceramides conversion to sphingosine and fatty acids by ASAH1. Ceramides are also converted to sphingomyelin during sphingolipid biosynthesis or generated during sphingolipid recycling. (**B**, **C**) Metabolomic analysis of the levels of ceramides, fatty acids, and sphingomyelin during replicative (**B**) or IR-induced (**C**) senescence. Dashed lines indicate p < 0.05. The x-axis indicates the categories and individual metabolites. Red bars indicate a log odds ratio (OR) > 1: an increased log odds of a higher metabolite concentration in the Replicatively senescent (**B**) or IR-induced senescent (**C**) group relative to the comparison group (Proliferating). Blue bars indicate a log odds ratio (OR) < 1: a decreased log odds of a higher metabolite concentration in the Replicatively senescent (**B**) or IR-induced senescent (**C**) group relative to the comparison group (Proliferating). Significant metabolites are highlighted in the table below each graph.

We then explored whether metabolites relevant to ASAH1 function – that is, ceramides, fatty acids and sphingomyelins – were altered in senescence (Figure 2A; results across all metabolites are included in Supplementary Table 2; ‘Replicatively Senescent vs Proliferating’ and ‘IR Senescent vs Proliferating’). When comparing replicatively senescent to proliferating control cells (Figure 2B) we found that one ceramide [Cer(d18:2/22:0)] and eight sphingomyelins [SM (OH) C16:1, SM C24:0, SM (OH) C24:1, SM C18:0, SM C24:1, SM (OH) C22:1, SM C26:1, SM (OH) C22:2] were significantly higher (log OR > 1; p < 0.05) in replicative senescence. When comparing IR-induced senescence to proliferating control cells (Figure 2C) we found that one ceramide [Cer(d18:1/24:1)] and two sphingomyelins [SM C16:0, SM C16:1] were significantly lower (log OR < 1; p < 0.05) in IR-induced senescence. Against our prediction, we did not observe an overlap or common changes in ASAH1-related metabolites between replicative and IR-induced senescence. The discrepancies could be dependent on the specific induction pathways, in this case replicative or IR-induced senescence, or heterogeneity of the senescent cells. Thus, these data suggest that while ASAH1-related metabolites may be altered in senescence, these two models of senescence do not share the metabolomic alterations.

### The rise in ASAH1 levels occurs largely independently of increased *ASAH1* mRNA stability or translation

**Figure 3:**
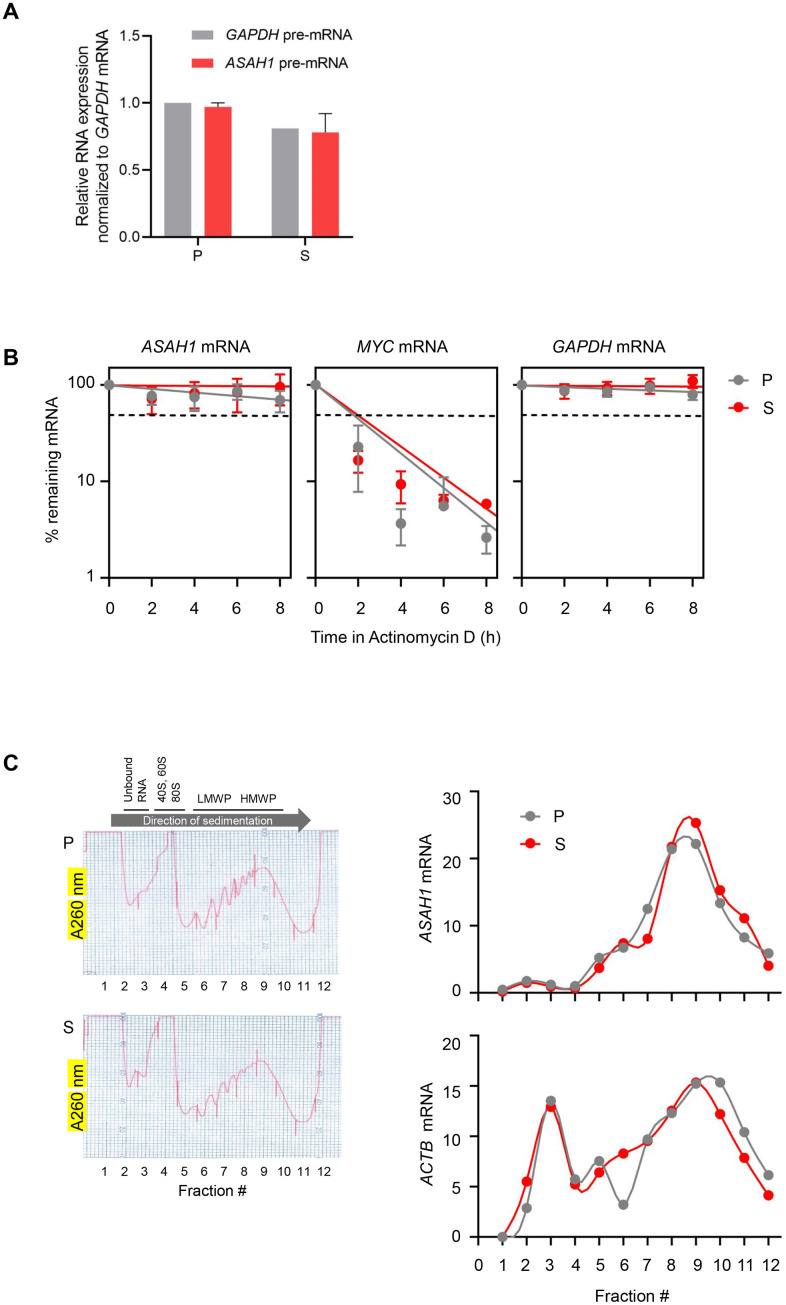
***ASAH1* mRNA stability and translation in senescent cells.** (**A**) RT-qPCR analysis of the levels of *ASAH1* and *GAPDH* pre-mRNAs using total RNA from proliferating (P) and senescent (S) cells upon replicative exhaustion and normalized to *GAPDH* mRNA levels. (**B**) Proliferating and senescent cells were treated with Actinomycin D for the times indicated to block transcription; total RNA was then isolated, and RT-qPCR analysis was performed to assess the levels of *ASAH1* mRNA. The labile *MYC* mRNA and the stable *GAPDH* mRNA were included as control transcripts. Discontinuous line indicates 50% of the original levels of mRNAs at time 0. (**C**) Cytoplasmic lysates obtained from P and S cells were fractionated through sucrose gradients to assess global polysome distribution profiles; ‘Unbound RNA’ fractions, fractions containing small ribosomal subunits (‘40S’), large ribosomal subunits (‘60S’), and monosomes (‘80S’), as well as polysomes of low and high molecular weight (LMWP and HMWP) are indicated. The relative distribution of *ASAH1* mRNA and housekeeping *ACTB* (*β-actin*) mRNA was studied by RT-qPCR analysis of RNA in each of 12 gradient fractions. Data in (**A**) and (**B**) represent the means and S.D. from three independent experiments; data in C are representative of three independent experiments.

To understand whether the twofold increase in *ASAH1* mRNA levels in senescent cells is due to a rise in transcription, we measured the levels of *ASAH1* pre-mRNA (a surrogate of *ASAH1* gene transcription) by RT-qPCR analysis using primers that amplify exon-intron junctions. As shown in Figure 3A, there were no significant changes in *ASAH1* pre-mRNA levels in senescent cells, suggesting that the increase in *ASAH1* mRNA levels in senescent cells was not due to elevated transcription (Figure 3A). To study if *ASAH1* mRNA was more stable in senescent cells upon replicative exhaustion, we treated fibroblasts with the RNA polymerase II inhibitor Actinomycin D in order to block transcription and measure by RT-qPCR analysis the rate of mRNA clearance (the half-life) in proliferating and senescent cells. As shown in Figure 3B, the *ASAH1* mRNA was very stable in both senescent and proliferating cells; the half-life appeared to be longer than 8 hours, and thus could not be measured by treatment with Actinomycin D, as this drug is quite toxic to cells beyond 8 hours of continuous exposure. As controls, we measured the decay of the labile *MYC* mRNA [32], and the stable *GAPDH* mRNA (Figure 3B). The relative levels of nuclear and cytoplasmic *ASAH1* mRNA were also unchanged with senescence (Supplementary Figure 2). We assayed the levels of noncoding RNAs *7SL* and *MALAT1* as cytoplasmic and nuclear controls, respectively (Supplementary Figure 2) [33, 34]. In sum, while *ASAH1* mRNA may be slightly more stable in senescent cells to account for the twofold rise in levels in senescent cultures, we were not able to accurately calculate the half-lives.

Next, we investigated the marked rise in ASAH1 protein levels. First, we tested whether ASAH1 translation may be elevated in senescent cells by fractionating the cytoplasmic compartment of the cell on sucrose gradients using standard procedures, as previously described [35]. We collected fractions that were devoid of ribosome particles (unbound RNA, fractions 1 and 2), fractions containing ribosomal subunits and single ribosomes (fractions 3-5), low-molecular-weight polysomes (LMWP, fractions 6-8), and high-molecular-weight polysomes (HMWP, fractions 9-12). RNA was prepared from each fraction, and the levels of *ASAH1* mRNA in each fraction were calculated as a percentage of total *ASAH1* mRNA in the gradient. This analysis revealed only a slight shift in the distribution of *ASAH1* mRNA across polysome fractions, suggesting that increased translation does not contribute strongly to the rise in ASAH1 protein levels in senescent cells (Figure 3C).

**Figure 4:**
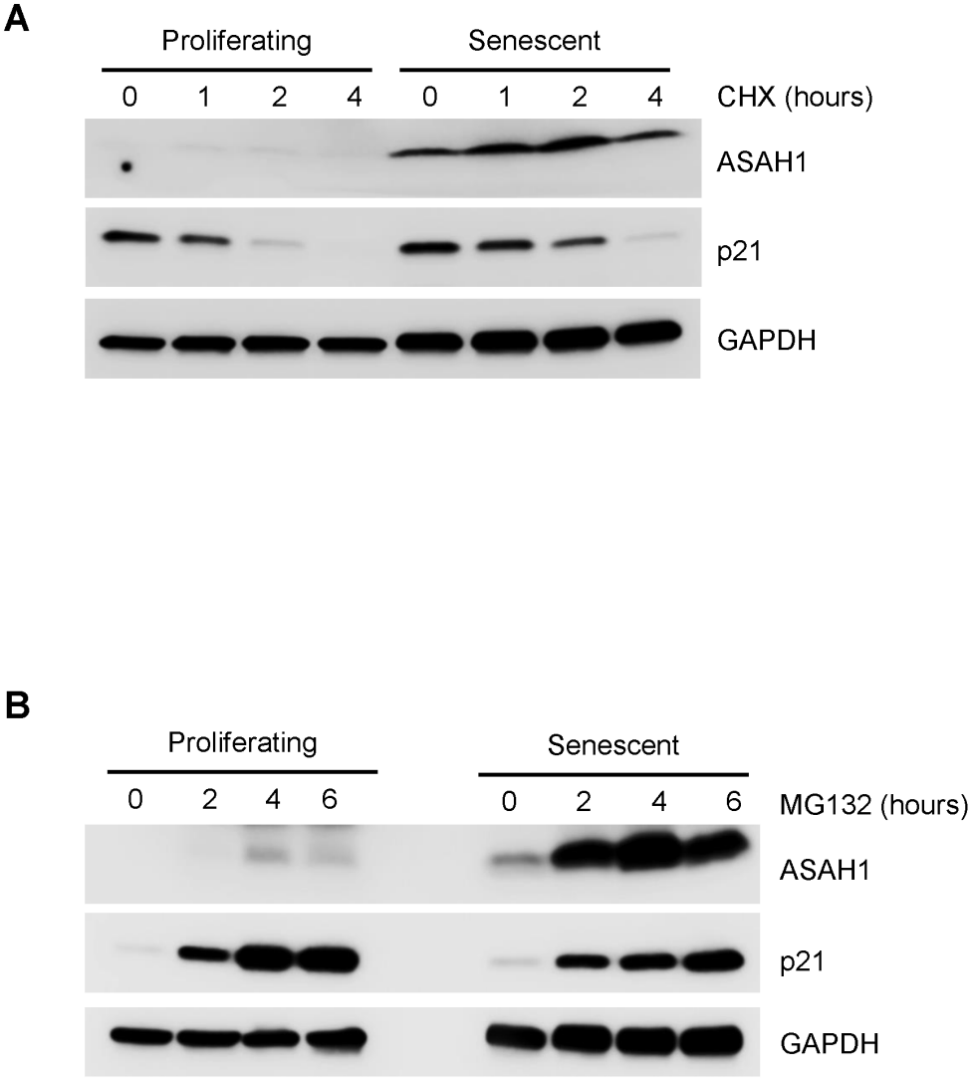
**Stability of the ASAH1 protein in proliferating and senescent cells.** (**A**) Western blot analysis of the levels of ASAH1 and loading control GAPDH in cells harvested after treatment with either vehicle (DMSO) or the protein synthesis inhibitor cycloheximide (CHX) for the times shown. (**B**) We assessed the levels of ASAH1 and control GAPDH in cells treated with either vehicle (ethanol) or 10 μM MG132 for indicated times by Western blot analysis. To monitor the efficiency of the treatments, we assessed the levels of the labile protein p21.

In the absence of clear evidence from polysome analysis that ASAH1 translation was elevated, we examined whether ASAH1 protein stability was altered between proliferating and senescent cells. To assess the changes in ASAH1 protein stability, we treated proliferating and senescent cells with cycloheximide (CHX), an inhibitor of *de novo* translation, and monitored the clearance of ASAH1 by Western blot analysis. Interestingly, ASAH1 was markedly stable in senescent cells, while it was undetected in proliferating cells; the control protein GAPDH was stable in both populations (Figure 4A). To look at ASAH1 protein stability in a different way, we treated cells with the proteasome inhibitor MG132. As shown in Figure 4B, ASAH1 protein was found to accumulate slightly in proliferating cells after four hours, but it was strongly induced after two hours of treatment with MG132 in senescent cells, as determined by Western blot analysis. We assessed the levels of the labile protein p21 to monitor the efficiency of the CHX and MG132 treatments (Figure 4A, 4B). Recently, it has been shown that ASAH1 is ubiquitinated and degraded by the proteasome through UBTD1 (ubiquitin domain-containing protein 1) [36]. These findings indicate that ASAH1 levels increase in senescent WI-38 fibroblasts largely due to enhanced protein stability.

### Loss of ASAH1 suppresses cellular senescence and alters associated metabolites

**Figure 5:**
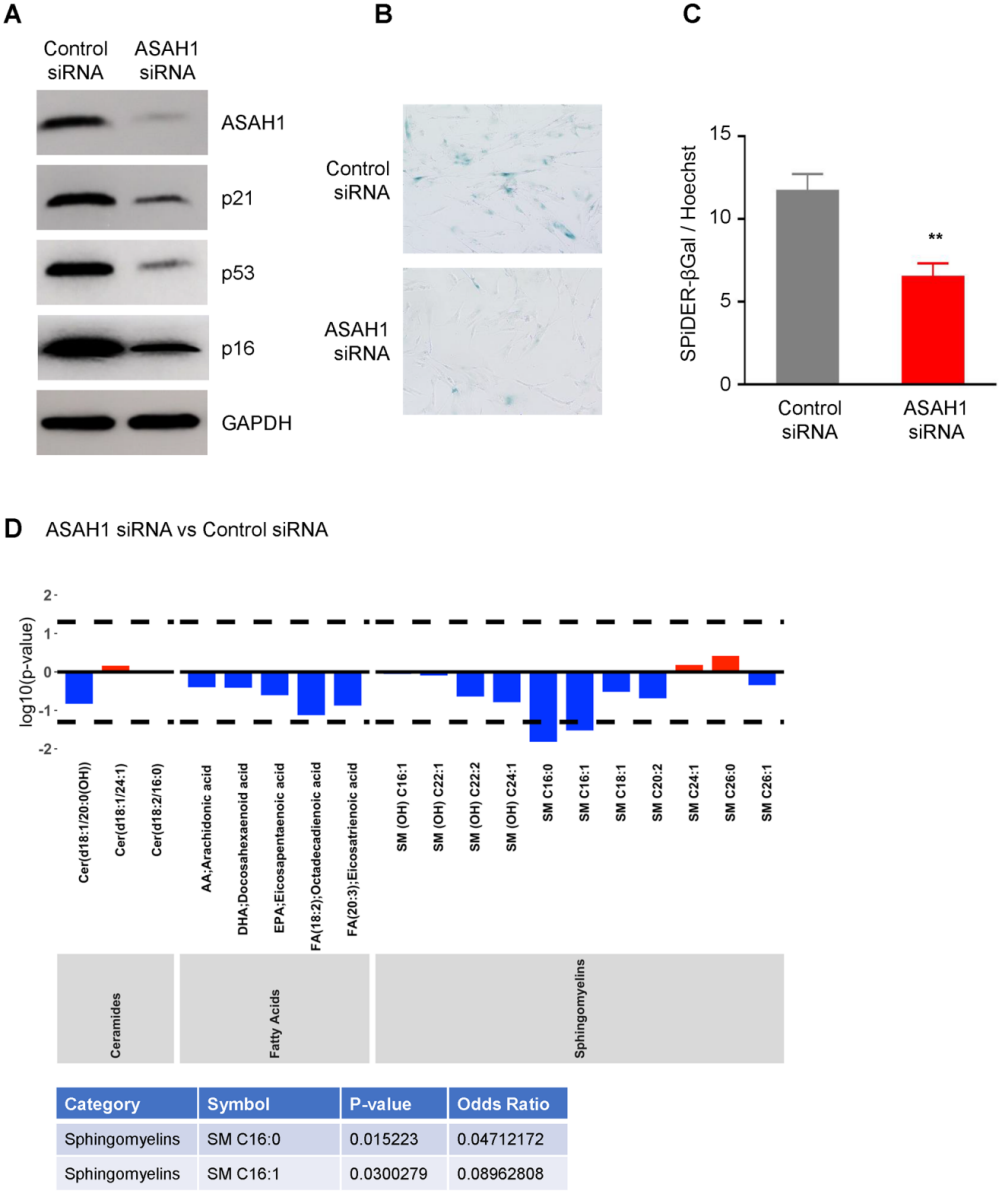
**Silencing ASAH1 represses senescence.** (**A**) Pre-senescent cells were transfected with either control or ASAH1 siRNA; 48 h after transfection cells were harvested and whole-cell lysates prepared for Western blot analysis to examine the levels of ASAH1, p16, p21, p53, and loading control GAPDH. (**B**, **C**) Detection of the senescent marker SA-βGal using traditional SA-βGal staining (**B**) and SPiDER-βGal (**C**) in the populations described in (**A**). (**D**) Metabolomic analysis of the levels of ceramides, fatty acids, and sphingomyelin after silencing ASAH1. Dashed lines indicate p < 0.05. The x-axis indicates the categories and individual metabolites. Red bars indicate a log odds ratio (OR) > 1: an increased log odds of a higher metabolite concentration in the ASAH1 group relative to the comparison group (Control siRNA). Blue bars indicate a log odds ratio (OR) < 1: a decreased log odds of a higher metabolite concentration in the ASAH1 group relative to the comparison group (Control siRNA). Significant metabolites are highlighted in the table below the graph.

To understand the role of ASAH1 in senescence, we transfected pre-senescent WI-38 cells with ASAH1-directed siRNA to reduce ASAH1 levels or a control siRNA. Silencing ASAH1 was confirmed by Western blot analysis, showing a robust reduction in cells transfected with ASAH1 siRNA compared to control cells (Figure 5A). ASAH1-depleted cells showed reduced levels of the senescence markers p16, p21, and p53 (Figure 5A). Furthermore, SA-βGal analysis revealed a decrease in positive (blue) senescent cells following ASAH1 depletion; this finding was confirmed by the SPiDER-βGal assay (Figure 5B, 5C), supporting the notion that lowering ASAH1 levels suppressed cell senescence. Cell cycle analysis indicated that depletion of ASAH1 in pre-senescent cells (PDL41) increased S-phase cells compared to control cells (Supplementary Figure 3) suggesting that ASAH1-depleted cells are more proliferative. When comparing ASAH1 siRNA to control siRNA populations (Figure 5D; results for all metabolites are in Supplementary Table 2 – ‘ASAH1 vs Control siRNA’) we found that sphingomyelins [SM C16:0, SM C16:1] were significantly decreased (log OR < 1; p < 0.05) in ASAH1-depleted cells. These changes may also be directly associated with ASAH1 silencing rather than senescence. Together, these data indicate that ASAH1 depletion reduces or delays cellular senescence and related metabolites, supporting a role for ASAH1 in promoting senescence.

### ASAH1 inhibition induces senescent cell death while ASAH1 depletion sensitizes cells to senolysis

**Figure 6:**
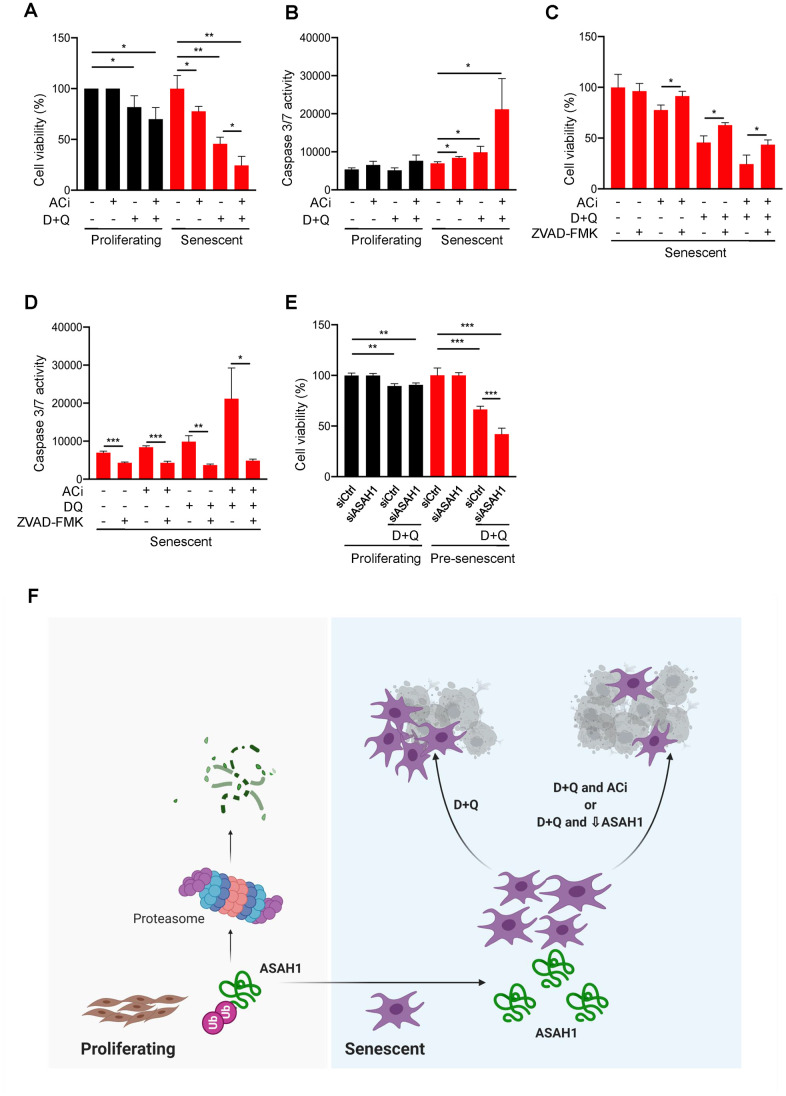
**ASAH1 is a potential senolytic target.** (**A**, **B**) Proliferating and senescent cells were either left untreated (DMSO) or treated with acid ceramidase inhibitor (ACi), with or without D+Q for 48 h, whereupon cell viability was assessed by direct cell counting (**A**) and the activities of caspase 3/7 were assessed (**B**). (**C**, **D**) Senescent cells were treated as in (**A**), with addition of ZVAD-FMK (a pan-caspase inhibitor) whereupon cell viability was assessed by direct cell counting (**C**) and caspase 3/7 activities were measured (**D**). (**E**) Proliferating or pre-senescent cells were transfected either with control or ASAH1 siRNA; two days after transfection, cells were treated with either control DMSO or D+Q for 48 h followed by assessment of cell viability by direct cell counting. (**F**) Proposed model: in proliferating cells, ASAH1 is rapidly degraded by machineries that include the ubiquitin-proteasome degradation system, while in senescent cells, ASAH1 is stable, in turn promoting senescent cell survival and resistance to senolytic cell death.

Senescence is detrimental for organ homeostasis with advancing age [37]. As senescent cells accumulate in aged tissue, they promote a range of age-related diseases and declines [38–40]; therefore, targeting and clearing senescent cells is clinically important. Senescent cell death has been achieved by a combination of Dasatinib plus Quercetin (D+Q), both approved by the US Food and Drug Administration (FDA) [41]. Thus, we examined whether ASAH1 might be implicated in senolysis and senescent-cell viability. To test these possibilities, we used two approaches. First, we treated proliferating or senescent cells with the ASAH1 inhibitor IV (ACi) [42] and monitored cell viability. As shown in Figure 6A, treatments with ACi alone did not have a significant impact on the viability of proliferating cells, but reduced by ~20% the viability of senescent cells. Importantly, we also assessed the effect of ACi in combination with D+Q, to evaluate whether ACi could enhance senolysis. Indeed, although the combination of ACi and D+Q was more toxic than D+Q alone for proliferating cells, it strikingly enhanced the effectiveness of D+Q in reducing senescent-cell viability (Figure 6A). To better characterize the increased death of senescent cells caused by ACi, we assessed apoptosis by monitoring caspase 3/7 activity. Inhibition of ASAH1 significantly enhanced caspase 3/7 activity in senescent cells treated with the senolytics D+Q (Figure 6B) in agreement with the reduction in viability observed in Figure 6A, while the different treatments did not influence caspase 3/7 activity in proliferating cells. To test if the reduction in viability caused by these drugs was due to apoptosis, we studied if the inhibitor of caspases ZVAD-FMK rescued cell death. As shown in Figure 6C, treatment with ZVAD-FMK significantly reduced senescent cell death by either by either D+Q alone or D+Q in combination with ACi (Figure 6C). The reduction of caspase activity in these conditions was confirmed by performing a caspase 3/7 activity assay (Figure 6D). These data suggest that pharmacological inhibition of ASAH1 potentiates cell death caused by senolytics at least in part by increasing caspase activity.

We then silenced ASAH1 in pre-senescent cells (PDL38-45) by transfection with ASAH1 siRNA and treated the cells with D+Q (see Materials and Methods). As shown, D+Q treatments decreased the number of viable proliferating and pre-senescent cells; however, pre-senescent cells were further sensitized to the toxicity of D+Q treatments if ASAH1 was inhibited, exhibiting more cell death than the untreated pre-senescent population (Figure 6E). Together, these data indicate that ASAH1 promotes senescent cell survival and resistance towards the senolytic combination D+Q (Figure 6F).

## DISCUSSION

The transcriptomic and proteomic analyses of WI-38 fibroblasts revealed that ASAH1 levels were highly elevated after induction of senescence by either replicative exhaustion or exposure to ionizing radiation. Validation of these results by RT-qPCR and Western blot analyses indicated that senescence increased *ASAH1* mRNA about twofold, while ASAH1 protein levels were strongly elevated (Figure 1). Senescent cells showed altered levels of ASAH1-related metabolites in replicative senescence and IR-induced senescence; these changes differed between the two senescence models (Figure 2), likely due to the fact that replicative senescence is more gradual, occurring after a lengthy time in culture that spans ~5 months, while IR-induced senescence is acute and is achieved in only 10 days. The absence of shared changes may also depend on the mode of senescence induction. It is also possible that IR triggered the hydrolysis of sphingomyelin to ceramide by a sphingomyelinase, as reported in bovine aortic endothelial cells (BAEC) [43]. The lack of significant changes in ceramides could be attributed to other conversions such as galactosylceramide, glucosideceramide or ceramide I phosphate [44, 45].

We dedicated extensive efforts towards finding the mechanism responsible for the rise in ASAH1 in senescent cells. We found no evidence for strong control via transcriptional upregulation, mRNA stabilization or enhanced translation (Figure 3), although we were not able to fully rule out if changes in *ASAH1* mRNA stability contributed to the enhanced expression of ASAH1 in senescence (Figure 3B). Instead, analysis of protein turnover indicated that ASAH1 is highly stable in senescent cells, while it is constitutively degraded in proliferating cells (Figure 4). In support of these findings, Torrino et al., recently proposed that ASAH1 can be degraded *via* the ubiquitin proteasome pathway through UBTD1 [36]. It will be important to test if UBTD levels or activity declines in senescent fibroblasts, although paradoxically, elevated UBTD1 was linked to cellular senescence through its ability to enhance the ubiquitination and degradation of MDM2 (a repressor of TP53) [46]. While these molecular specifics await further study, we propose that the striking increase in ASAH1 abundance was largely due to enhanced ASAH1 protein stability in senescent cells. Further studies are needed to delineate in molecular detail the ubiquitination of ASAH1, the lysine residues modified, the ubiquitin ligase responsible, and the signaling pathway that might control the ubiquitination of ASAH1.

The accumulation of senescent cells in tissues and organs has been linked to the pathogenesis and severity of age-associated diseases such as cancer, arthritis, cataracts, neurodegeneration, and atherosclerosis [47]. This accumulation is associated with metabolic reprogramming in senescent cells towards specific activities like elevated production of reactive oxygen species, the SASP trait, and lysosomal biogenesis [48, 49]. As mentioned above, the lysosomal ASAH1 converts ceramides to fatty acids and sphingosine, which is then converted to sphingosine 1 phosphate. These metabolites play crucial roles in cellular processes like proliferation, differentiation, and survival [50]. Accordingly, failure to maintain these metabolites in balance was associated with several age-associated conditions including cancer and Alzheimer’s disease, making ASAH1 an attractive therapeutic target [25, 51, 52]. Given the growing interest in selectively eliminating senescent cells, we examined if depletion of ASAH1 altered senescence traits. We found that silencing ASAH1 decreased SA-βGal activity, lowered senescence markers p16, p21, and p53, and reduced the concentration of ASAH1-related metabolites (sphingomyelins) as compared to control cells (Figure 5). These findings suggest that ASAH1 may directly contribute to maintaining the senescence program and its depletion may either delay senescence or cause a shift towards other processes such as cell death. Additionally, reduced levels of ASAH1 may reverse the metabolic alterations observed in senescence.

ASAH1 has been linked to several malignancies, including glioblastoma, acute myeloid leukemia, melanoma, prostate cancer, and colon cancer [53–56]. Thus, ASAH1 inhibitors are gaining interest in cancer therapy and were found to trigger apoptosis in cancer cells; in this regard, ASAH1 inhibitors were shown to be effective in killing glioblastoma cancer stem cells as well as head and neck cancer cells (HNC) [57, 58]. Therefore, we questioned whether ASAH1 plays a role in senescent cell survival, whether it can potentially be a target of senolysis, and whether inhibition of ASAH1 using ACi may be cytotoxic for senescent cells. Indeed, ACi did induce senescent cell death (Figure 6A). In line with this interesting effect, deletion of ASAH1 using CRISPR-Cas9 in A375 melanoma cells was found to disrupt ceramide metabolism and directed cells towards either apoptosis or senescence [59], suggesting that ASAH1 may potentially be a therapeutic target for both cancer and senescence. It is worth noting that ACi enhanced senescent cell death and caspase 3/7 activity, which was further augmented in combination with D+Q (Figure 6A–6D). In an effort to exploit ASAH1 as a pharmacological target, several inhibitors have been developed for cancer therapeutics [60]. Whether these or other novel ASAH1 inhibitors influence senescent cell survival in clinical settings remains to be tested.

It has been shown that ASAH1 promotes resistance of cancer cells to radiation therapy, and depletion of ASAH1 using RNAi improved the therapeutic response [61]. Thus, we tested if depletion of ASAH1 influenced the efficacy of D+Q treatments. Strikingly, ASAH1-depleted cells were more susceptible to death when treated with D+Q than cells expressing normal ASAH1 levels (Figure 6). While the activity of caspases 3/7 was initially increased in ASAH1 depleted cells, it was further enhanced by the D+Q treatments (Figure 6B). These data suggest that ASAH1 promotes senescent cell resistance to senolytics and its depletion improved the efficacy of the senolytics D+Q, likely due to the elevated activity of caspases (Figure 6B). In sum, we report here that ASAH1 is rapidly degraded in proliferating cells, while in senescent cells it remains stable and can promote cell survival and resistance to senolytics (Figure 6F). The roles of ASAH1 in senescence-associated processes such as inflammation and angiogenesis warrant future investigation. In closing, a more comprehensive understanding of the metabolic changes driven by ASAH1 across senescence models is also needed in order to fully elucidate the value of ASAH1 in eliminating senescent cells and in sensitizing senescent cells towards the cytotoxic impact of established senolytics.

## Supplementary Material

Supplementary Figures

Supplementary Table 1

Supplementary Table 2
