# Supplementary material for: Acid ceramidase promotes senescent cell survival
Source: Aging (Albany NY). 2021 Jun 8;13(12):15750–69. doi: 10.18632/aging.203170 (PMC8266329; doi:10.18632/aging.203170)
Supplement: Supplementary Figures [file aging-13-203170-s001.pdf]

SUPPLEMENTARY FIGURES

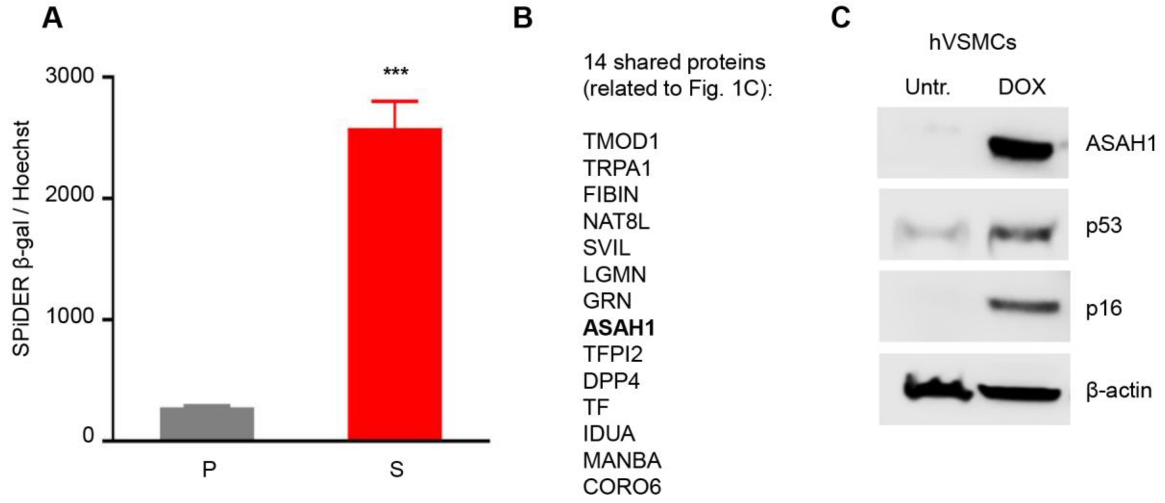

Supplementary Figure 1. ASAH1 is highly expressed in senescent cells. (**A**) SA β-gal activity is shown for proliferating (PDL 20) (P) and replicative senescent (PDL 47) (S) WI-38 cells using the SPiDER β-gal activity assay (Dojindo). (**B**) The 14 shared proteins that are up-regulated in senescent WI-38 cells in both replicative and IR-induced senescent models from proteomics analysis in which the corresponding mRNAs are also upregulated, from the RNA-sequencing data. (**C**) Human vascular smooth muscle cells (hVSMCs) were rendered senescent by exposure to doxorubicin (DOX) and the levels of ASAH1 and the senescent markers p53 and p16 were assessed by Western blot analysis. The levels of β-actin were monitored as a loading control.

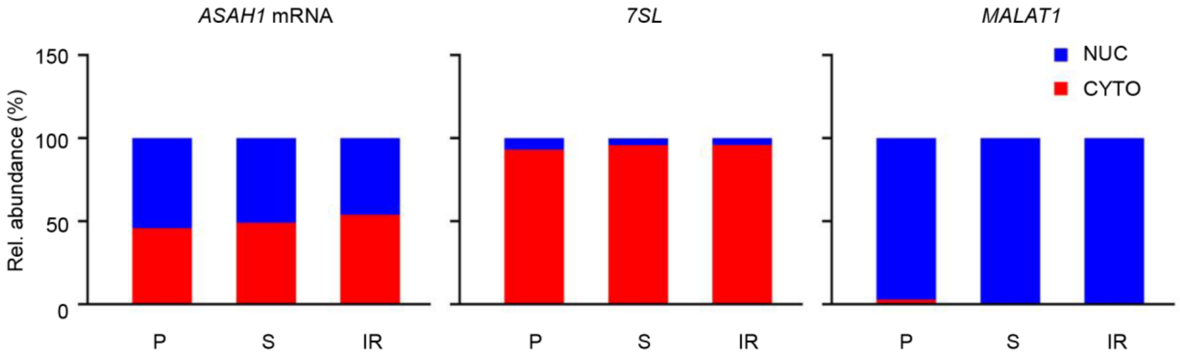

Supplementary Figure 2. ASAH1 mRNA levels in nuclear and cytosolic compartments. The levels of *ASAH1* mRNA in the nucleus (blue) and cytosol (red) were assessed by RT-qPCR analysis in proliferating WI-38 cells (P), WI-38 cells rendered senescent by replicative exhaustion (S), and WI-38 cells rendered senescent by IR (IR). *7SL* and *MALAT1* were used as controls for the cytosol and nucleus, respectively.

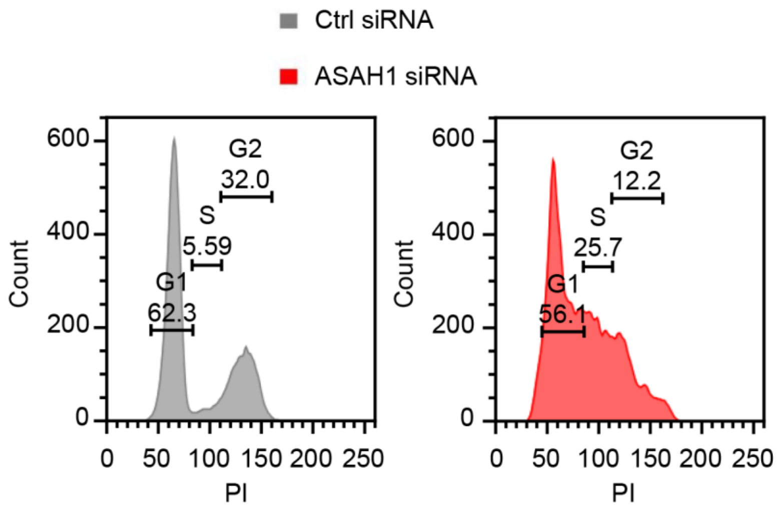

Supplementary Figure 3. Cell cycle distribution analysis after ASAH1 depletion in pre-senescent cells. WI-38 cells rendered pre-senescent by passage to PDL41 were transfected with either control (gray) or ASAH1 (red) siRNA. Forty-eight hours later, propidium iodide (PI) was incorporated into these cells to measure cell cycle distribution by flow cytometry. The percentages of cell cycle phases G1, S, and G2 are displayed on the graphs.
